# Kuntai Capsule Combined With Letrozole on Gonadal Hormone Levels and Ovarian Function in Patients With PCOS: A Systematic Review and Meta-Analysis

**DOI:** 10.3389/fendo.2021.789909

**Published:** 2021-12-28

**Authors:** Xing Tang, Qinwan Huang, Chengcheng Wang, Da Zhang, Shoujin Dong, Congcong Yu

**Affiliations:** ^1^ Sichuan Integrative Medicine Hospital, Chengdu, China; ^2^ Sichuan Institute for Translational Chinese Medicine, Chengdu, China; ^3^ Sub-Health Clinical Detection and Evaluation Laboratory, Chengdu, China; ^4^ Chengdu University of Traditional Chinese Medicine (TCM), Chengdu, China; ^5^ Chengdu Medical College, Chengdu, China; ^6^ Respiratory Department, Chengdu First People’s Hospital, Chengdu, China; ^7^ The University of British Columbia (UBC) Centre for Heart Lung Innovation, St. Paul’s Hospital, Vancouver, BC, Canada

**Keywords:** Kuntai capsule, letrozole, polycystic ovary syndrome, meta-analysis, gonadal hormone levels, ovarian function

## Abstract

**Background:**

The efficacy of Kuntai capsule combined with letrozole (LE) in improving ovarian function of polycystic ovary syndrome (PCOS) has been evaluated before, but there is still a lack of evidence-based support for the regulation of sex hormone levels. In recent years, new randomized clinical trials (RCTs) have been reported on the effect of combined therapy on regulating sex hormone levels.

**Objective:**

We aimed to systematically evaluate the efficacy of Kuntai capsule combined with LE in the treatment of PCOS.

**Methods:**

A search across the China Biomedical Literature Database (CBM), China National Knowledge Infrastructure (CNKI), China Science and Technology Journal Database (VIP), Wanfang database, PubMed, Web of Science, The Cochrane Library, and Embase was conducted on Kuntai capsule combined with LE in the treatment of PCOS. The time of the self-built database was up to April 30, 2021. RCTs of LE in the control group and LE combined with Kuntai capsule in the experimental group were selected. RevMan5.3 software was used for data analysis.

**Results:**

A total of 17 studies were gathered, which included 1,684 patients. The meta-analysis results showed that the total effective rate of the combined group was 93.36% and that of the LE group was 78.15%. The improvement in the ovulation rate, pregnancy rate, number of mature follicles, endometrial thickness, cervical mucus score, and serum follicle stimulating hormone (FSH), luteinizing hormone (LH), and prolactin (PRL) in the combined group was consistent with the results of a previous meta-analysis and was better than that in the LE group (*p* < 0.05). In addition, the combination group was better than the LE group in regulating the levels of estradiol (E2) and testosterone (T) (*p* < 0.05). There were no adverse drug reactions in the two groups during treatment.

**Conclusion:**

As a type of pure traditional Chinese medicine preparation, Kuntai capsule combined with LE had a better effect than LE alone in the treatment of PCOS, with advantages mainly reflected in enhancing ovarian function and regulating the levels of sex hormones *in vivo*, among others, but the value of combined therapy still needs to be verified by more high-quality RCTs.

## 1 Introduction

Kuntai capsule is the first Chinese patent medicine approved for the treatment of ovarian function decline (1). Kuntai capsule can improve the ovarian function in polycystic ovary syndrome (PCOS), and its efficacy is mainly manifested in improving the ovulation rate, pregnancy rate, number of mature follicles, endometrial thickness, cervical mucus score, etc. (2) Kuntai capsule uses the theory of traditional Chinese medicine to open up the cell vein, promote blood circulation, remove stasis, replenish blood, and clear collaterals so as to effectively improve endometrial blood flow and improve ovarian and uterine hormone secretion. The pharmacological action of letrozole (LE) is to reduce estrogen production by inhibiting aromatase. In premenopausal women, inhibition of estrogen synthesis leads to increased gonadotropin [luteinizing hormone (LH) and follicle stimulating hormone (FSH)] levels, which stimulate follicular growth, leading to ovulation (3–5). However, a large number of clinical application studies have found that, due to the short half-life of the drug, its effect continues to decrease when the estrogen level decreases in the late follicular development, and the dominant follicle may not be discharged. Various studies have shown that the success rate of LE for ovulation induction is about 70%–84%, but the pregnancy rate is only 20%–27% (6). Whether the combination of Kuntai capsule and LE can exhibit a synergistic effect needs support from large-sample evidence-based medical validation. By looking for evidence-based medical proof and combining expert clinical drug use experience, expert consensus can finally be reached, and guidelines will be formed and issued for clinical frontline doctors in the use of Kuntai capsule combined with LE. This is of great significance for reducing clinical risks and improving clinical efficacy. In this paper, randomized clinical trials related to the combined intervention of two drugs on PCOS were screened from multiple databases, aiming to evaluate the clinical value of Kuntai capsule combined with LE in the intervention of PCOS and to provide new evidence for further research on the synergistic and antagonistic effects of Kuntai capsule combined with LE in clinical applications.

## 2 Materials and Methods

### 2.1 Retrieval Strategy

Multiple Chinese and English databases were searched using a computer, including PubMed, Web of Science, EMBASE, Cochrane Library, China Biomedical Literature Database (CBM), China National Knowledge Infrastructure (CNKI), Wanfang Database, and China Science and Technology Journal Database (VIP). The last time all databases were searched was updated to April 1, 2021. English search adopted medical subject headings (MeSH) term and free word database and applied the following terms: “Polycystic ovary syndrome”, “Kuntai Capsule”, and “Letrozole”. The Chinese search criteria were as follows: “duonangluanchaozonghezheng”, “kuntaijiaonang”, and “laiquzuo”.

### 2.2 Inclusion Criteria

1. Participants: participants meet the clear diagnostic criteria for PCOS (e.g., the Rotterdam Diagnostic Criteria in 2003 or the diagnostic criteria issued and implemented by the Ministry of Health of China in 2011);
2. Intervention: Kuntai capsule combined with LE;
3. Comparison: LE alone;
4. Outcomes: change in the ovulation rate, pregnancy rate, total effective rate, number of mature follicles, endometrial thickness, cervical mucus score, FSH, LH, prolactin (PRL), estradiol (E2), and testosterone (T) after the treatment;
5. Study design: randomized clinical trial (RCT); and
6. Language: English or Chinese studies.

### 2.3 Exclusion Criteria

1. Non-RCT articles, review articles, animal experiments, etc.;
2. The research content does not conform to the theme; and
3. The subjects had other diseases and medications that might affect the statistical results.

### 2.4 Literature Screening, Data Extraction, and Quality Assessment

Literature screening, data extraction, and quality assessment were carried out independently by two researchers back to back, and the final results were cross-checked.

If there was any disagreement, two people discussed it; if no agreement could be reached, a third party made the decision. All search results retrieved from each database were imported into the EndNote literature management software. Firstly, duplicate studies were eliminated, and then the title and abstract were read for preliminary screening. Finally, the full text was read and a final decision was made according to the inclusion and exclusion criteria. Excel tables were established for data extraction, including the first author and year of publication, sample size, age, course of the disease or years of infertility, intervention measures, course of treatment, and outcome indicators. The modified Jadad scale was used for quality assessment. Studies scored 4–7 were classified as high-quality studies, while those with scores 1–3 were considered as low-quality studies.

### 2.5 Statistical Methods

The outcome index data of the included studies were input into RevMan 5.3 software for heterogeneity test and meta-analysis. Among them, the odds ratio (OR) was used for the statistical analysis of counting indicators; standardized mean difference (SMD) was used for the statistical analysis of quantitative indicators. The 95% confidence intervals (CIs) were calculated for both types of data. The heterogeneity of each outcome index was tested using the *Q* series and *I* ^2^ methods. When the heterogeneity was small (*p* > 0.1, *I* ^2^ < 50%), the fixed effects model was used for analysis. When the heterogeneity was large (*p* ≤ 0.1, *I* ^2^ ≥ 50%), the random effects model was used for analysis.

### 2.6 Prognostic Criteria

1. Cure: menstruation and sex hormone levels return to normal;
2. Significant effect: normal menstrual period, sparse menstrual volume, and acne and sebum overflow symptoms were significantly improved;
3. Effective: sparse menstrual volume, acne and sebum overflow symptoms have been partially improved;
4. Ineffective: patients with amenorrhea, acne, and sebum overflow symptoms show no significant improvement or even aggravation;

Total effective rate=[(cured cases+significant effective cases+effective cases)/total cases]×100%.

## 3 Results

### 3.1 Literature Retrieval Results

**Figure 1:**
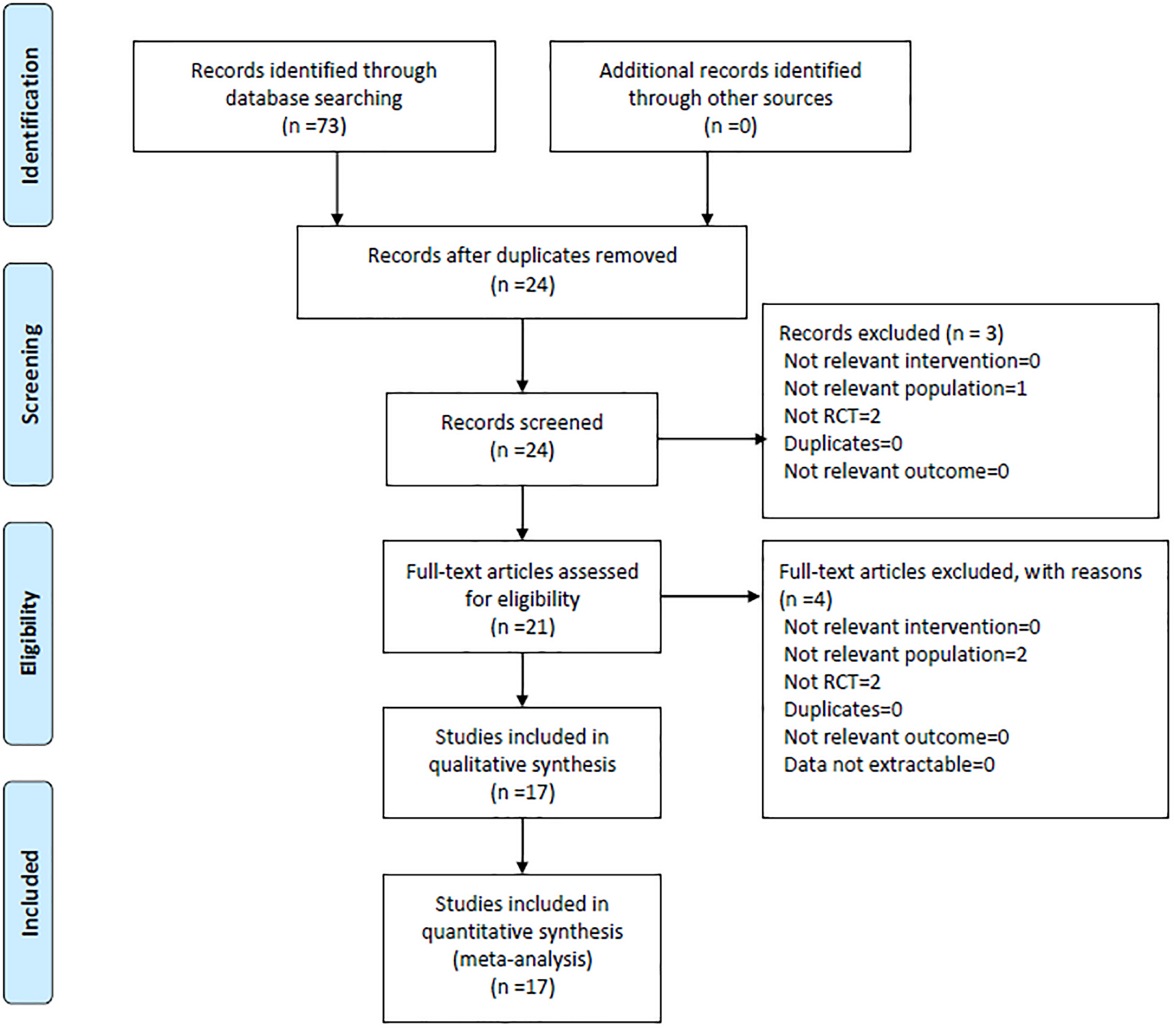
Flow diagram of the study selection process.

Through the databases, 73 studies in Chinese and 0 study in English were retrieved. Among them, 49 were duplicate studies and 24 were obtained after deletion. By reading the abstracts, 1 study that was not consistent with the research content and 2 studies that were not RCTs were excluded. After reading the full texts, 2 articles not consistent with the research content and 2 articles that were not RCTs were excluded. Finally, all the remaining 17 studies (7–23) were included in the meta-analysis, with publication years from 2016 to 2020, including 1,684 patients. The basic characteristics of the included studies are shown in **Table 1**. The study selection was summarized in a PRISMA flow diagram (**Figure 1**).

**Table 1:** Basic features of the included studies.

Study	Year	Language	Age (years), $\bar{x}$ ± *S*		No. of patients (intervention/comparison)	Weight (kg) or BMI (kg/m^2^), $\bar{x}$ ± *S*		Trial group	Control group	Time of taking medicine	Duration	Outcome	Jadad score
			Trial group	Control group		Trial group	Control group						
Fan and Xue (7)	2020	Chinese	33.96 ± 8.12	33.21 ± 7.38	46/46	Weight: 57.11 ± 9.89	Weight: 56.24 ± 9.78	Kuntai capsule (2 g, tid po) + letrozole	Letrozole (2.5 mg qd po), 5 days	Begins on the 5th day of menstruation	84 days	AGHIJKL	3
Guo (8)	2020	Chinese	28.42 ± 8.07	27.3 ± 9.57	38/38	Not mentioned	Not mentioned	Kuntai capsule (2 g, tid po) + letrozole	Letrozole (2.5 mg qd po), 5 days	Begins on the 3rd day of menstruation	5 menstrual cycles	DEGJK	3
Li (9)	2020	Chinese	34.56 ± 4.12	34.75 ± 3.51	50/50	BMI: 23.91 ± 2.15	BMI:24.03 ± 1.93	Kuntai capsule (2 g, tid po) + letrozole	Letrozole (2.5 mg, qd po), 5 days	Menstruation begins on the 5th day	90 days	BCDEFJ	3
Zhang (10)	2020	Chinese	29.26 ± 2.28	29.34 ± 2.17	56/55	BMI: 55.63 ± 8.16	BMI: 55.59 ± 8.21	Kuntai capsule (2 g, tid po) + letrozole	Letrozole (2.5 mg, qd po), 5 days	Menstruation begins on the 5th day	50 days	A	3
Chen (11)	2019	Chinese	34.5 ± 3.2	32.5 ± 3.6	40/40	Not mentioned	Not mentioned	Kuntai capsule (2 g, tid po) + letrozole	Letrozole (2.5 mg, qd po), 5 days	Menstruation begins on the 5th day	50 days	DEGHL	4
Chen (12)	2017	Chinese	36.5 ± 1.7	36.5 ± 1.7.	40/40	Not mentioned	Not mentioned	Kuntai capsule (2 g, tid po) + letrozole	Letrozole (2.5 mg, qd po), 7 days	Menstruation begins on the 5th day	50 days	ADEF	3
Chen (13)	2016	Chinese	25.9 ± 3.5	26.2 ± 3.8	16/16	BMI: 27.1 ± 4.3	BMI: 27.4 ± 4.2	Kuntai capsule (2 g, tid po) + letrozole	Letrozole (2.5 mg, qd po), 5 days	Menstruation begins on the 5th day	Until pregnancy or menstruation	BCDE	3
Liu (14)	2019	Chinese	28.75 ± 2.59	29.17 ± 2.66	46/46	Not mentioned	Not mentioned	Kuntai capsule (2 g, tid po) + letrozole	Letrozole (2.5 mg, qd po), 5 days	Menstruation begins on the 5th day	30 days	ABCGHJK	3
Wang (15)	2019	Chinese	31.04 ± 3.21	31.23 ± 3.09	48/48	Not mentioned	Not mentioned	Kuntai capsule (2 g, tid po) + letrozole	Letrozole (2.5 mg, qd po), 5 days	Menstruation begins on the 5th day	90 days	ADEF	4
Wang et al. (16)	2018	Chinese	20–35	19–28	55/50	BMI: 20–35	BMI: 19–28	Kuntai capsule (2 g, tid po) + letrozole	Letrozole (2.5 mg, qd po), 5 days	Menstruation begins on the 5th day	Until pregnancy or menstruation	CDEF	3
Wu (17)	2016	Chinese	26.4 ± 3.2	26.3 ± 3.1	85/85	Not mentioned	Not mentioned	Kuntai capsule (2 g, tid po) + letrozole	Letrozole (2.5 mg, qd po), 5 days	Menstruation begins on the 5th day	28 days	CDE	3
Yang et al. (18)	2019	Chinese	29.59 ± 8.12	29.74 ± 7.19	55/55	Not mentioned	Not mentioned	Kuntai capsule (2 g, tid po) + letrozole	Letrozole (2.5 mg, qd po), 7 days	Menstruation begins on the 5th day	21 days	BCE	4
Yang and Huang (19)	2016	Chinese	36.4 ± 2.3	36.2 ± 2.1	35/35	Not mentioned	Not mentioned	Kuntai capsule (2 g, tid po) + letrozole	Letrozole (2.5 mg qd po), 7 days	Menstruation begins on the 5th day	50 days	ADEFGHIJKL	4
Yang and Zhou (20)	2016	Chinese	26.44 ± 3.67	26.53 ± 3.77	82/80	BMI: 20.75 ± 2. 22	BMI: 20. 65 ± 2. 10	Kuntai capsule (2 g, tid po) + letrozole	Letrozole (2.5 mg qd po), 5 days	Menstruation begins on the 5th day	28 days	CDE	3
Yu et al. (21)	2018	Chinese	29.42 ± 2.34	29.11 ± 2.28	42/42	Weight: 56.21 ± 8.90	Weight: 56.86 ± 8.44	Kuntai capsule (2 g tid po) + letrozole	Letrozole (2.5 mg qd po), 5 days	Menstruation begins on the 5th day	30 days	BCDEFGHIJKL	3
Zhang (22)	2018	Chinese	28.35 ± 3.12	28.18 ± 3.88	52/52	Not mentioned	Not mentioned	Kuntai capsule (2 g tid po) + letrozole	Letrozole (2.5 mg, qd po), 5–7 days	Menstruation begins on the 5th day	56 days	CDE	3
Zhong et al. (23)	2018	Chinese	No data	No data	60/60	Not mentioned	Not mentioned	Kuntai capsule (2 g, tid po) + letrozole	Letrozole (2.5 mg, qd po), 5 days	Menstruation begins on the 5th day	50 days	DEGHL	3

Outcomes: A, total efficiency; B, ovulation rate; C, pregnancy rate; D, number of mature follicles; E, endometrial thickness; F, cervical mucus score; G, serum follicle stimulating hormone; H, luteinizing hormone; I, hurried, milk element (PRL); J, estradiol; K, testosterone; L, progesterone.

### 3.2 Ovulation Rate and Cycle Pregnancy Rate

#### 3.2.1 Ovulation Rate

**Figure 2:**
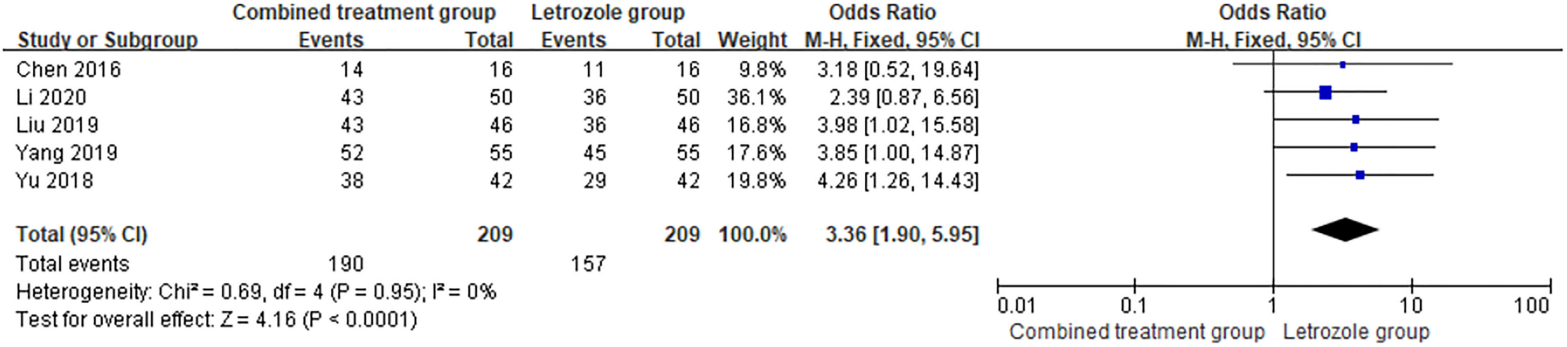
Meta-analysis of the effect of Kuntai capsule combined with letrozole on the ovulation rate in polycystic ovary syndrome (PCOS).

Ovulation rate was reported in 5 studies (9, 13, 14, 18, 21), with 209 cases in both the combined group and the LE group, and there was no heterogeneity between the studies (*p* = 0.95, *I* ^2^ = 0%). The fixed effects model was selected for meta-analysis, and the results showed that the difference between the two groups was statistically significant (OR = 3.36, 95%CI = 1.90–5.94, *p* < 0.0001). It showed that the combination therapy can improve the ovulation rate of patients. The forest diagram is shown in **Figure 2**.

#### 3.2.2 Pregnancy Rate

**Figure 3:**
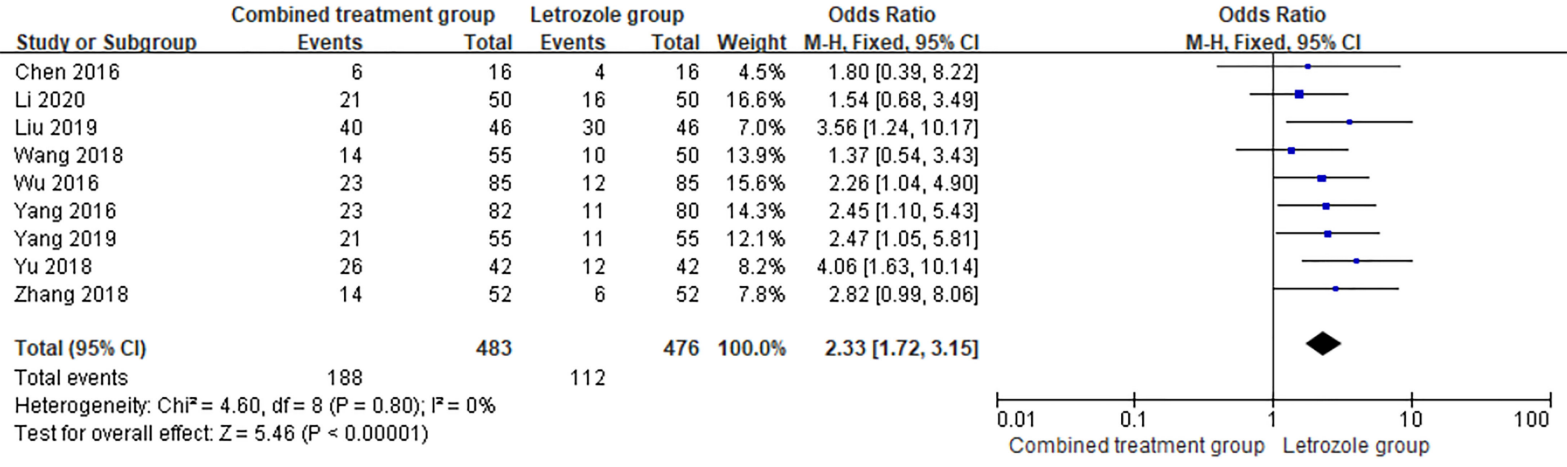
Meta-analysis of the effect of Kuntai capsule combined with letrozole on the pregnancy rate in polycystic ovary syndrome (PCOS).

Pregnancy rate was reported in 9 studies (9, 13, 14, 16–19, 21, 22), which included 483 patients and 188 pregnancies in the combination group, with a pregnancy rate of 38.92%. Of the 476 patients in LE group, 112 were pregnant, and the pregnancy rate in the LE group was 23.53%. There was no heterogeneity between the studies (*p* = 0.80, *I* ^2^ = 0%). The fixed effects model was selected for meta-analysis, and the results showed that the differences between the two groups were significant (OR = 2.33, 95%CI = 1.72–3.15, *p* < 0.00001). The pregnancy rate of the combined group was higher than that of the LE group, indicating that the combined use of Kuntai capsule can increase the pregnancy rate. The forest map is shown in **Figure 3**.

#### 3.2.3 Total Effective Rate

**Figure 4:**
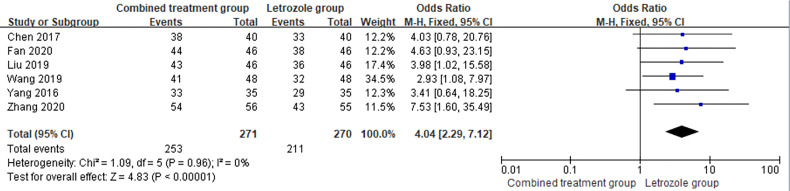
Meta-analysis of the effect of Kuntai capsule combined with letrozole on the total effective rate in polycystic ovary syndrome (PCOS).

Total effective rate was reported in 6 studies (7, 10, 12, 14, 15, 19), including 271 cases in the combined group and 253 cases which were effective. The total effective rate was 93.36% in the combined group. Of the 270 cases in LE group, 211 were effective. The total effective rate in the LE group was 78.15%. There was no heterogeneity among studies (*p* = 0.96, *I* ^2^ = 0%). The fixed effects model was selected for meta-analysis, and the results showed that the total response rate for Kuntai capsule combined with LE in the treatment of PCOS increased with statistical significance compared with that for letrozole alone (OR = 4.04, 95%CI = 2.29–7.12, *p* < 0.00001). The forest map is shown in **Figure 4**.

### 3.3 Indicators Related to Follicular Maturation

The observed data of ovulation after treatment included the number of mature follicles, endometrial thickness on ovulation day, and the cervical mucus score, which showed high heterogeneity (*I* ^2^ = 93%). After sensitivity analysis, the heterogeneity was reduced after one study was removed, but it still existed. The heterogeneity test results of the last three indicators indicated that there was heterogeneity among the indicators, and so the random effects model was selected for meta-analysis. The results showed that the number of mature follicles, endometrial thickness on ovulation day, and the cervical mucus scores of the combined group were better than those of the LE group. The results of the indicators related to follicular maturation are shown in **Table 2**.

**Table 2:** Results of indicators related to follicular maturation.

Indicators	No. of included studies	No. of patients (intervention/comparison)	Heterogeneity test	SMD (95%CI)	*p*-value
Number of mature follicles	13 (7–9, 11–13, 15–19, 21, 22)	643/636	*p* < 0.01, *I* ^2^ = 93%	0.97 (0.72–1.21)	<0.00001
Endometrial thickness on ovulation day	14 (7–9, 11–19, 21, 22)	698/691	*p* < 0.01, *I* ^2^ = 93%	1.80 (1.32–2.28)	<0.00001
Cervical mucus score	6 (8, 11, 12, 15, 17, 22)	270/265	*p* < 0.01, *I* ^2^ = 93%	3.19 (1.92–4.47)	<0.00001

SMD, standardized mean difference.

### 3.4 Serum Sex Hormone Levels

#### 3.4.1 Follicle Stimulating Hormone

**Figure 5:**
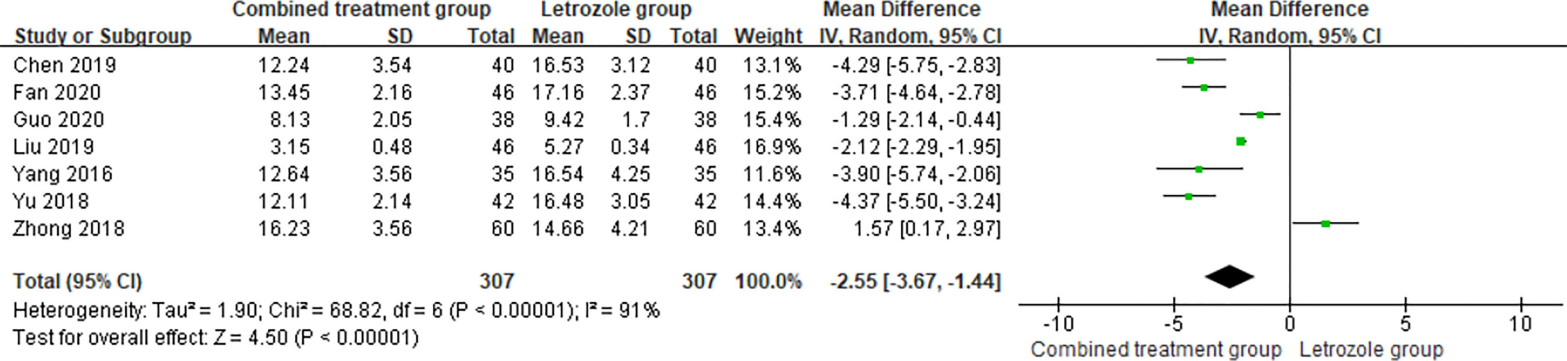
Meta-analysis of the effect of Kuntai capsule combined with letrozole on serum follicle stimulating hormone (FSH) in polycystic ovary syndrome (PCOS) treatment.

The time of venous blood collection measured by FSH ranged from 2 to 4 days from the beginning of the menstrual cycle. The results of the heterogeneity test suggested that there was heterogeneity in the FSH levels between studies, so the random effects model was selected for meta-analysis. The results showed that there was a significant difference in the FSH levels between the two groups (*p* < 0.05), as shown in **Figure 5**.

#### 3.4.2 Luteinizing Hormone

**Figure 6:**
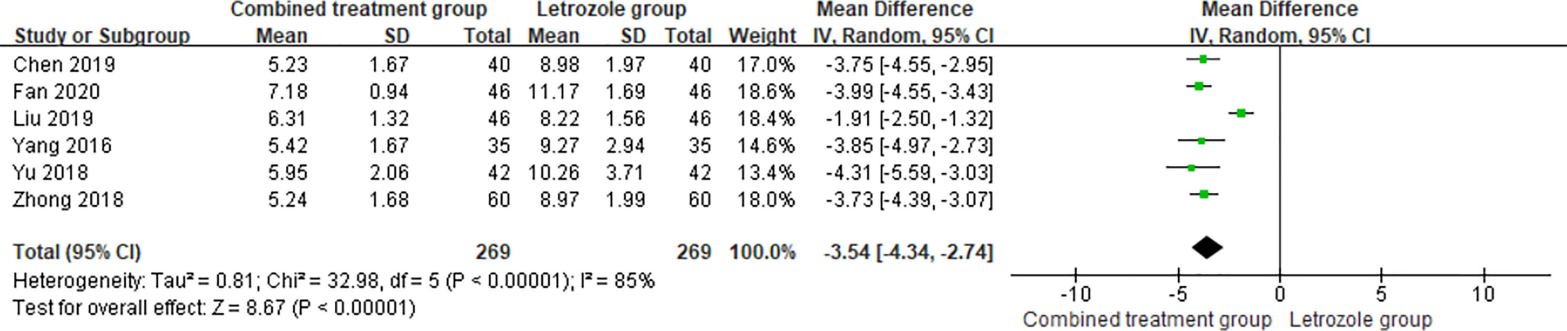
Meta-analysis of the effect of Kuntai capsule combined with letrozole on luteinizing hormone (LH) in polycystic ovary syndrome (PCOS).

The time of venous blood collection measured by LH ranged from 2 to 4 days from the beginning of the menstrual cycle. The results of the heterogeneity test suggested that there was heterogeneity in the LH levels between studies, so the random effects model was selected for meta-analysis. The results showed significant differences between the two groups (*p* < 0.05), as shown in **Figure 6**.

#### 3.4.3 Prolactin

**Figure 7:**
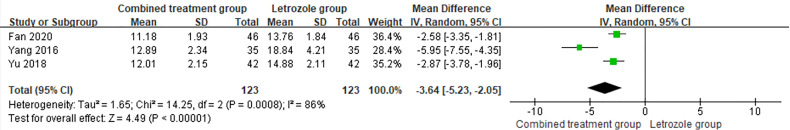
Meta-analysis of the effect of Kuntai capsule combined with letrozole on prolactin (PRL) in the treatment of polycystic ovary syndrome (PCOS).

The time of venous blood collection measured by PRL ranged from 2 to 4 days from the beginning of the menstrual cycle. The heterogeneity test results suggested that the PRL levels were heterogeneous between studies, so the random effects model was selected for meta-analysis. The results showed that PRL was significantly different between the two groups (*p* < 0.05), as shown in **Figure 7**.

#### 3.4.4 Estradiol

**Figure 8:**
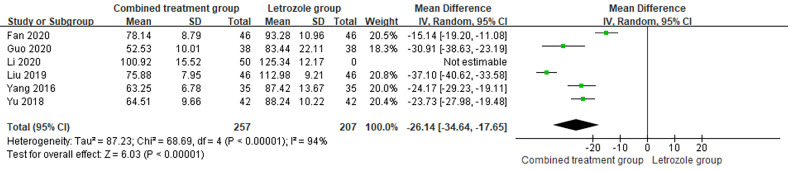
Meta-analysis of the effect of Kuntai capsule combined with letrozole on estradiol (E2) in polycystic ovary syndrome (PCOS) treatment.

The time of venous blood collection measured by E2 ranged from 2 to 4 days from the beginning of the menstrual cycle. The heterogeneity test results suggested heterogeneity of the E2 levels between studies, so the random effects model was selected for meta-analysis. The results showed significant differences in E2 levels between the two groups (*p* < 0.05), as shown in **Figure 8**.

#### 3.4.5 Testosterone

**Figure 9:**
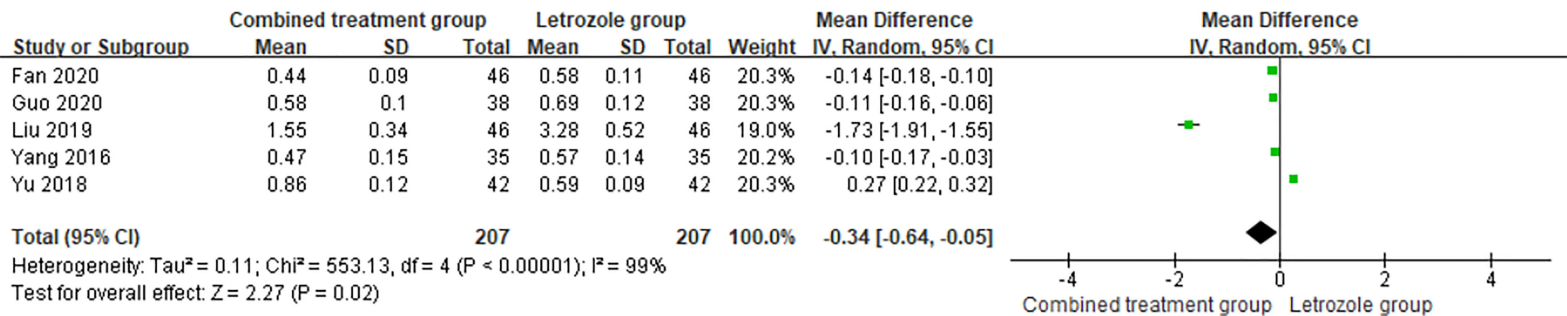
Meta-analysis of the effect of Kuntai capsule combined with letrozole on testosterone (T) in the treatment of polycystic ovary syndrome (PCOS).

The time of venous blood collection measured by T ranged from 2 to 4 days from the beginning of the menstrual cycle. The heterogeneity test results suggested heterogeneity in the T levels between studies, so the random effects model was selected for meta-analysis. The results showed significant differences in T levels between the two groups (*p* < 0.05), as shown in **Figure 9**.

#### 3.4.6 Progesterone

**Figure 10:**
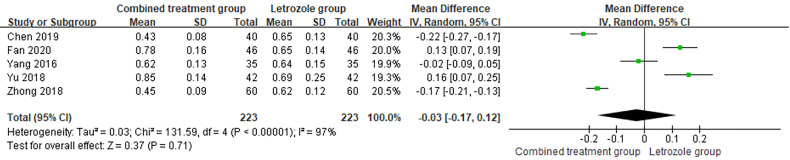
Meta-analysis of the effect of Kuntai capsule combined with letrozole on progesterone (P) in polycystic ovary syndrome (PCOS) treatment.

The time of venous blood collection measured by P ranged from 2 to 4 days from the beginning of the menstrual cycle. The heterogeneity test results suggested heterogeneity in the P levels between studies, so the random effects model was selected for meta-analysis. The results showed no significant difference in the levels of P between the two groups (*p* > 0.05), as shown in **Figure 10**.

## 4 Discussion

### 4.1 Innovations and Limitations of This Study

In this study, 17 RCTs (7–23) on Kuntai capsule combined with LE in the treatment of PCOS were included. All the included studies were originally published in Chinese; therefore, this meta-analysis assists in allowing the information from these studies to reach a broader range of readers by summarizing the major findings in English. The meta-analysis results showed that the total effective rate, ovulation rate, and pregnancy rate in the combined group were higher than those in the LE group, and the number of mature follicles, endometrial thickness, and the cervical mucus scores were better than those in the LE group. Meanwhile, the levels of FSH, LH, PRL, E2, and T decreased in the combined group, but there was no significant difference in the *p*-values. Kuntai capsule, as a new preparation, has a certain role in improving the ovarian microenvironment, which can strengthen the function of the ovary, promote ovulation, and improve the pregnancy rate, and has plant estrogen-like effects, which can effectively adjust the sex hormone levels in the body, improve the blood flow state and receptive endometrium, promote endometrial hyperplasia, and provide a higher possibility for embryo implantation. Compared with other traditional biological indicators, the anti-Müllerian hormone (AMH) has several obvious advantages in the evaluation of ovarian reserve. As the most accurate biomarker of ovarian aging, AMH can reflect the decline trend of ovarian reserve with age earlier, and its level is not affected by the menstrual cycle, hormonal contraceptives, and pregnancy (24). None of the 17 included studies detected AMH indicators. In future clinical trials, more attention should be paid to the regulation of Kuntai capsule combined with LE on AMH.

### 4.2 Comparison Between Previous Studies and This Study

A meta-analysis on the efficacy and feasibility of Chinese patent medicine combined with LE in the treatment of female ovulation disorders has recently been published in Front. Pharmacol. (https://doi.org/10.3389/fphar.2021.722122) (25). This paper analyzed the advantages and disadvantages of six kinds of Chinese patent medicines in the treatment of ovulation disorders, namely, Kuntai capsules, Fuke Zaizao capsules, Fufang Xuanju capsules, Fuke Yangying capsules, Bailing capsules, and Dingkun Dan. This study has a number of strengths. We aimed to reach expert consensus by seeking evidence-based medical proof and combining experts’ clinical experience in drug use to form and issue guidelines for clinical frontline doctors on the use of proprietary Chinese medicines. It is of great significance to reduce clinical risks and improve clinical efficacy. This study also has limitations. Firstly, the number of articles about Kuntai capsule was the largest among the included studies, but there were no special and detailed analyses on its efficacy. In addition to the study of Kuntai capsule, the remaining five proprietary Chinese medicines were included in very few studies. Therefore, the evidence for conclusions based on network meta-analysis is low. Secondly, only three outcome measures were considered, namely, ovulation rate, pregnancy rate, and endometrial thickness on the day the follicles matured. The studies did not mention the regulatory effect of LE combined with Kuntai capsule on various hormone levels.

Clinical studies on interventions using Kuntai capsule combined with LE for PCOS are gradually increasing. A total of 13 RCTs were published before 2019 (26), and 4 additional RCTs (7–10) were published in 2020, an increase of 30.77%, indicating that Kuntai capsule combined with LE has gradually become a hotspot of clinical research in recent years. A previous meta-analysis (26) systematically evaluated the efficacy of a two-drug intervention for PCOS, but due to the number of cases and the quality of the study, there may be a risk of bias. In addition, a previous meta-analysis (26) focused more on the efficacy of the combination of the two drugs in improving ovarian function (ovulation rate, pregnancy rate, number of mature follicles, endometrial thickness, and cervical mucus score), but did not carry out detailed analysis on the efficacy in regulating sex hormone levels. In 2020, there were 379 new cases included in 4 RCTS (7–10), an increase of 29.04% compared with that in previous meta-analysis (6) (1,305 cases). All 4 studies (7–10) mentioned random method (random number table method), and 3 of them (7–9) analyzed the comparability of baseline data. Three studies (7–9) carried out detailed analysis on the changes of sex hormone levels before and after intervention, with complete outcome data. A previous study (26) found that there was no significant difference in the efficacy of LE alone in regulating P, E2, and T between the two drugs combined with LE (*p* > 0.05). However, this study found that Kuntai capsule combined with LE also had significant effects on regulating the levels of E2 and T (*p* < 0.05), indicating that the combination of the two drugs had a synergistic effect on regulating the levels of sex hormones. During the treatment period, no adverse reactions such as nausea, vomiting, dizziness, headache, breast pain, and skin allergy occurred in the combination and the LE group, indicating that the combination of the two drugs did not produce significant antagonistic effects and did not increase the incidence of adverse reactions. The newly included literature provides higher quality evidence to support the efficacy of the two-drug therapy in PCOS intervention.

### 4.3 Possible Pharmacological Effects of Kuntai Capsule and Letrozole on Ovulation Induction

PCOS is an endocrine disorder syndrome with the coexistence of reproductive dysfunction and abnormal glucose metabolism (27). The pathological physiological manifestations of the disease are mainly on the regulation of the hypothalamus–pituitary–ovarian axis dysfunction and the gonadotropin-releasing hormone secretion of the hypothalamus, which can lead to the secretion of PRL, FSH, and LH by the pituitary gland, thus increasing ovarian secretion of T and estrogen and inhibiting the development of follicles, eventually causing follicular atresia, being unable to escape from the ovaries. This leads to secondary amenorrhea and infertility. In addition, due to the lack of periodic progesterone secretion of PCOS, the endometrium is stimulated by pure estrogen for a long time, and endometrial dysplasia is prone to occur, which is not conducive to the implantation of fertilized eggs. Therefore, the key to the treatment of PCOS is to effectively regulate the hormone levels of patients, restore their ovulation function, and enhance the receptivity of the endometrium. For patients with fertility needs, modern medicine usually adopts symptomatic treatment by inducing ovulation. According to traditional Chinese medicine, the etiology and pathogenesis of PCOS are closely related to kidney deficiency, liver stagnation, spleen deficiency, cuspidor, and blood stasis. The formulation of Kuntai capsule was adapted from the “Treatise on Typhoid and Miscellaneous Diseases” by Zhang Zhongjing (28), which included six traditional Chinese medicines: Radix Scutellariae, *Rehmannia glutinosa*, Coptis Rhizoma, *Paeonia lactiflora*, Donkey Jiao, and *Poria cocos*. The king medicine, cooked Dihuang, can produce lean pulp, nourish the yin and kidney, and nourish the blood. *Rhizoma coptidis* and *Paeonia lactiflora* play the role of soothing liver and stopping spasmolysis, clearing heat and dampness; donkey-hide gelatin and cooked rehmannia glutinosa have a synergistic effect on nourishing Yin and blood. The adjuvant Astragalus dispels heat and reduces fire, and *Poria cocos* invigorates the spleen and tranquilizes the mind. The combination of various drugs can nourish the yin, clear heat, calm the mind and eliminate irritability, help in the communication between the heart and kidney, regulate yin and yang, and cure both symptoms and root causes (29).

As a pure Chinese medicine preparation, Kuntai capsule combined with LE can adjust the hormone levels, improve ovarian function, increase the ovulation rate, confer good cervical mucus characteristics of sperm, which is conducive to the thickening of the cervix and endometrium, create favorable conditions for embryo implantation, and improve the infertility pregnancy rate of patients with PCOS (30). In order to provide more reliable and rigorous evidence-based support for the combined clinical use of the two drugs, higher quality, large sample size, and multicenter clinical trials are needed for verification, and the follow-up time should be extended to increase the recording of pregnancy outcomes. Therefore, long-term analysis and evaluation of Kuntai capsule combined with LE in the treatment of PCOS are needed.

## Data Availability Statement

The original contributions presented in the study are included in the article/supplementary material. Further inquiries can be directed to the corresponding author.

## Author Contributions

XT was the guarantor of the article. XT and CW drafted the manuscript. XT and SD developed the search strategy. XT and DZ independently screened the potential studies and extracted the data. CY and QH edited the manuscript. CY arbitrated any disagreement and ensured that no errors occurred during the review. All authors critically reviewed, revised, and approved the subsequent and final version of the protocol.

## Funding

This study is supported by the National Key Research and Development Program of China (no. 2018YFC1704701) and the Science and Technology Research Special Project of Sichuan Administration of Traditional Chinese Medicine (no. 2020LC0201).

## Conflict of Interest

The authors declare that the research was conducted in the absence of any commercial or financial relationships that could be construed as a potential conflict of interest.

## Publisher’s Note

All claims expressed in this article are solely those of the authors and do not necessarily represent those of their affiliated organizations, or those of the publisher, the editors and the reviewers. Any product that may be evaluated in this article, or claim that may be made by its manufacturer, is not guaranteed or endorsed by the publisher.
